# Accuracy of a novel method for IOP measurement without applying pressure on the cornea

**DOI:** 10.1007/s10792-024-03213-7

**Published:** 2024-07-01

**Authors:** Amit Biran, Mordehai Sayar, Moshe Lazzar, Michael Mimouni, Itzchak Beiran

**Affiliations:** 1 Department of Ophthalmology, Soraski Medical Center, Tel Aviv, Israel; 2 Department of Applied Physics, Weizmann Institute of Science, Rehovot, Israel; 3 Department of Ophthalmology, Rambam Health Care Campus Affiliated With the Bruce and Ruth Rappaport Faculty of Medicine, Technion-Israel Institute of Technology, POB 9602, 3109601 Haifa, Israel

**Keywords:** Intraocular pressure, Device, Non touch

## Abstract

**Purpose:**

To assess the accuracy of a prototype novel instrument for intra ocular pressure (IOP) measurements not involving corneal pressure application.

**Design:**

Prospective case control study.

**Methods:**

An institutional study including 16 healthy volunteers without ocular pathology. IOP in both eyes of the participants was measured four times in different body positions with the novel prototype and reference instrument (Goldmann applanation tonometer (GAT) or iCare (iCare Finland OY, Vantaa, Finland)). IOP results were compared between the prototype and the reference instruments in 116 pairs of measurement.

**Results:**

Overall no statistically significant difference was found between the presented prototype and the reference instrument. Stratifying measurements by instrument used revealed no significant difference for GAT and statistical significant (yet clinically insignificant) difference for iCare.

**Conclusions:**

The presented prototype demonstrates good clinical agreement of IOP measuring results with reference instruments Further large-scale studies assessing this instrument in glaucoma patients are warranted.

**Supplementary Information:**

The online version contains supplementary material available at 10.1007/s10792-024-03213-7.

## Introduction

Increased intra ocular pressure (IOP) is the single most important risk factor for the development of primary open angle glaucoma (POAG) [1]. All treatment modalities used for POAG as well as Normal tension glaucoma (NTG) are aimed at decreasing IOP [2, 3].

Measuring IOP is a key factor in appreciating the status of glaucoma and decision making in the treatment of the disease. Classically, measuring IOP is performed at the ophthalmologist's office using the Goldmann applanation tonometer (GAT) [4].

There are two major limitations in this practice. First, the measurement reflects the IOP value at a given moment and IOP is known to fluctuate during the day. Therefore, a single office measurement does not necessarily reflect the maximal pressure value which may dictate treatment decision making [5, 6]. Secondly, performing the measurement necessitates a professional medical team member (ophthalmologist or ophthalmic technician) to perform.

Alternative instruments for IOP measurements include non-contact “puff” tonometry which is based on an air jet directed at the cornea. The force of the air jet increases rapidly and linearly and the measured time taking the jet to flatten the central cornea is inversely related to the resistance of the cornea representing the IOP. Aside from simpler operation this instrument suffers the same limitations of GAT as far as need for operator (less qualified but still needed) and a single non representative measurement [7]. The Tonopen (Medtronic, Buffalo, NY, USA) and iCare (iCare Finland OY, Vantaa, Finland). allow for IOP measurement in scarred corneas or in patients unable to sit in front of the slit lamp, however, they involve direct contact with the cornea, require professional operators and do not allow for repeated non office/nonhospital measurements [7].

To get a better and more reliable picture of the relevant IOP multiple measurements during the day are needed. Using a day curve which consists of multiple daily measurements provides a better appreciation of actual IOP but is highly inconvenient for both the patient and the examiner because of the need for repetitive in-office examinations by an ophthalmic professional. Self iCare measurement enabling the patient to take their own IOP is aimed at offering a solution to the need for continuous measurements throughout the day. However, this instrument is based on contact measurement using a disposable probe and as such is hard to perform and has not gained much popularity [8].

Non-contact tonometry (= measuring IOP without physical contact with the cornea) reduces the risk of infection, does not require the use of local anesthetic and can be carried out by an inexperienced examiner or untrained professional [9]. Good correlation of IOP measurements performed by table mounted “puff” tonometer and portable non-contact tonometers (Pulsair and ORA) with GAT measurement results were previously reported [10–12]^.^

All the above mentioned IOP measuring devices (contact & non-contact) rely upon the counter force needed to be applied to the cornea to equalize the internal pressure posed on its inner surface by the IOP [13]. Corneal biomechanical properties influences the IOP measurement results (9). In one of the portable non-contact instruments (ORA) at attempt to measure those properties and correct the IOP result accordingly was made. No concensus exists as to the validity of these “corrected” results [14].

Based on the above mentioned data it is reasonable to look for a non-contact, cornea independent IOP measurement.

A different and novel approach for a non-contact, cornea independent IOP measurement relates to the change in pupil's area or diameter as a possible representor of IOP. If the resistance to the movement of the iris posed by the anterior chamber fluid is IOP dependent—the higher the IOP the higher the resistance—then the rate of pupil area change in response to ambient light may indicate the IOP. In that case, measuring pupil’s area change rate in response to standard change in environmental illumination can serve to calculate the IOP.

The goal of the present study was to evaluate the precision of a new non-contact optical measurement of IOP based on pupillary area change analysis. We assumed a correlation between the IOP and pupil area change. The proposed evaluation instrument (prototype) measures the area of the pupil and the rate of pupil dilatation after a flash blue light illumination based on an offline calculation of the recorded area measurements over time.

## Materials and methods

A new optical prototype system was developed to measure IOP and its measurements were compared to GAT (Hagg- Streit, Bern) or iCare (iCare ic200, iCare, Finland OY) readings.

The post illumination pupil response (PIPR) amplitude was found to be largest with 1-s short wavelength pulses (482 nm ~ 12.8 log quanta/cm^2^ Sec) and 6-s PIPR with the least intra- and inter-individual difference (CV < / = 0.2) [15]. Based on this data, the test conducted included pupil dark accommodation for a period of 10 s followed by a 1.5 Sec. diffused blue light illumination (λ = 480 nm). The constriction and dilation of the pupil were documented.

The IOP testing prototype (patent pending) is composed of a near infra-red (NIR) binocular camera including two blue light LEDs and diffusers. The camera and the blue light illumination are controlled by a laptop computer using proprietary software. The binocular camera uses NIR light to record pupil dynamics at a rate of 13 images per second. The software module allows human–machine interface, blue light illumination time control (1.5 Sec) as well as video recording of both eyes’ images for offline data processing (Supplementary Fig. 1).

As iris performance is patient specific the instrument must be calibrated for each user. In the present study, the first measurement served to calibrate the prototype based on the “gold standard” value with the GAT measurement. At the following measurements, IOP was compared between prototype results based on pupil dilatation rate and GAT/iCare measurements at the same time and circumstances.

The prototype has been tested by the Standards Institution of Israel (SII) according to ISO 15004–2:2007. The prototype is classified as Group 1 for NIR light and Group 2 for the blue light.

The study was approved by the institutional review board (IRB) for human clinical trials of Rambam medical center IRB approval No. 0137–19. Written informed consent for the research was obtained from each participant.

### Comparative test procedure

Healthy volunteers not suffering from any known eye pathology were recruited. To make the comparison for variable IOP values, measurements in lying position aiming at increasing the IOP were included. Thus, for each participant four comparative IOP tests were performed in a dark room with dimmed lights as follows:

- In sitting position by the IOP testing prototype and immediately afterwards by GAT.
- After 2 min in flat supine position, by the IOP testing prototype and immediately afterwards by iCare.
- After additional 2 min in lying position with legs raised 45 degrees, by the IOP testing prototype and immediately afterwards by iCare.
- After additional 2 min in sitting position by the IOP testing prototype and immediately afterwards by GAT.

The primary measurement (a) served for calibration of the prototype while the other measurements were independent and were compared to the GAT/iCare results. The primary measurements were excluded from the statistical analysis.

All measurements were taken between 10 AM and 1230 PM.

Measurements were taken first by the prototype and then by the reference instrument in order to prevent possible “ocular massage effect” [16]

### Statistical analysis

Descriptive statistics in terms of median and 25–75% (IQR) was calculated for all study parameters. Wilcoxon Signed Ranks Test was used to evaluate the IOP measurement difference between the prototype and the standard devices. Repeated measure analysis evaluated the variation in IOP measurement difference between study groups b–d (supine, lying with raised legs, sitting). *P* < 0.05 was considered significant. SPSS version 27 was used for statistical analysis.

Suggested strategy to avoid multi co-llinearity if two variables are highly correlated (typically above 0.8 or − 0.8) is creating a new variable that combines information from taking the average, sum, or other appropriate combination [17].

In order to avoid possible effect of multi-co-linearity on the study results we have compared the results of the right eyes to the left eyes in the prototype and the reference instrument in each of the three measurements' conditions. In five out of six set of measurements no statistical significance was found (*P* value between 0.11 and 0.76). In the prototype measurement in the lying position a borderline statistical significance of 0.047 was demonstrated (with mean values of 18 mmHg in the right eye and 18.6 mmHg in the left eye for this set of measurements which means no clinical relevance of this difference). These results exclude the influence of multi-co-linearity regarding the inclusion of both eyes in the present study.

## Results

Of 20 patients three were unable to comprehend and cooperate with the testing and Data was not obtained for one participant and one eye of another participant. Out of 16 participants (31 eyes) whose tests were included in this report 4 were unable to lie with their legs lifted in 45 degrees and hence had only 3 measurements (sitting, supine, sitting).

A total of 116 pairs of measurements were documented. The 31 first round of sitting measurements served for examinee-specific calibration. Out of the 85 pairs of measurements who were compared 31 were taken by iCare in supine position, 23 by iCare in lying position with raised legs and 31 by GAT in sitting position.

**Fig. 1:**
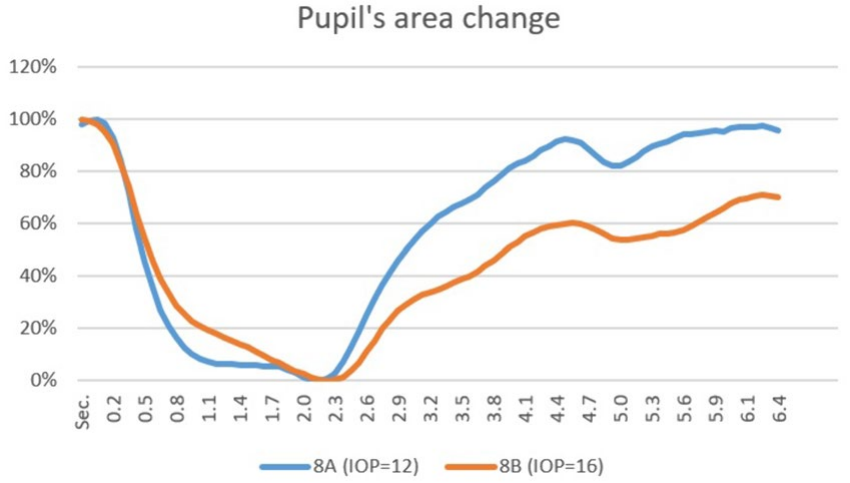
pupil’s area change: “natural course” of pupil’s area change in percentage from dark value following light flash at IOP of 12 & 16 mmHg

The Fig. 1 presents the “natural course” of pupil’s area changes during the test at two different IOP levels (12 and 16 mmHg). IOP values ranged between 9 and 23 mmHg. The difference in mean IOP value between the highest and lowest values in the three measuring groups was 2 mmHg.

**Fig. 2:**
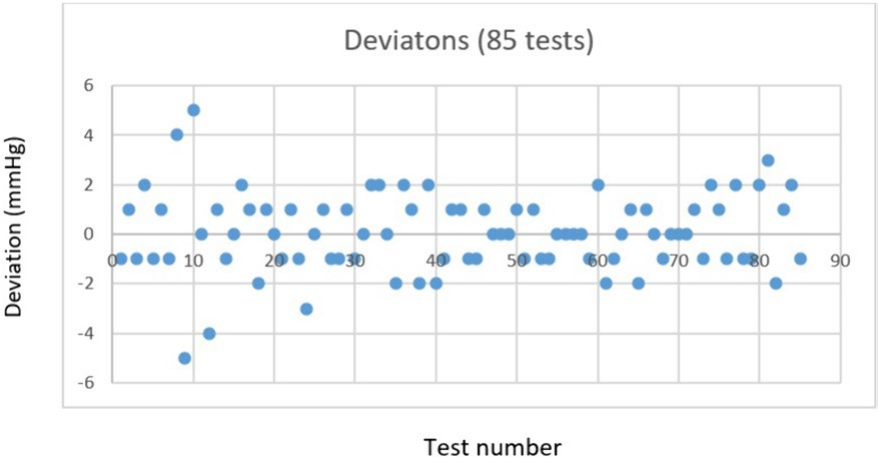
intra ocular pressure difference between prototype & reference: IOP measurement Deviation distribution between prototype & reference

The Fig. 2 presents the distribution of the difference between the measurements taken with reference instrument (iCare or GAT) and the prototype.

**Fig. 3:**
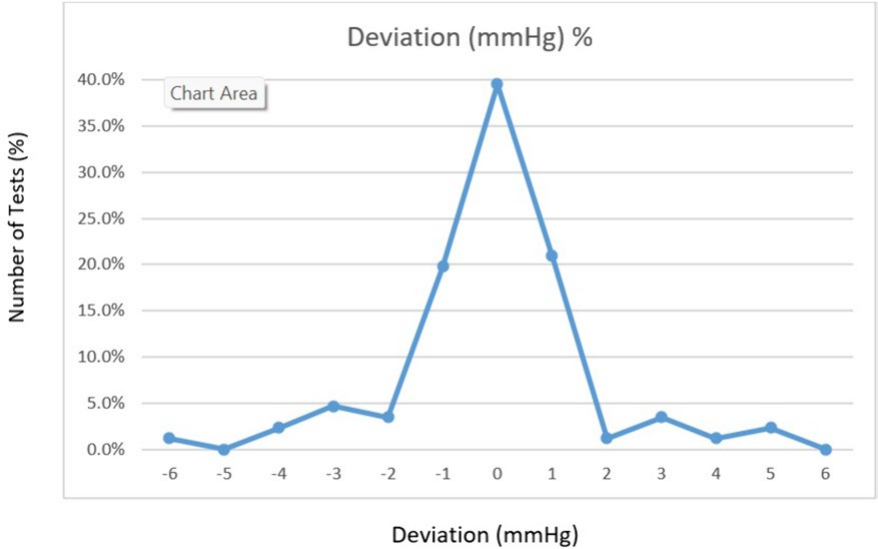
distribution of difference in IOP: distribution of the difference in IOP values between the prototype and the reference instrument

There were no significant differences between prototype and standard reference in terms of mean IOP in all tests (18.2 ± 2.6 mmHg versus 18.3 ± 2.4 mmHg, *p* = 0.76) or sitting position (18.4 ± 2.3 mmHg versus 18.2 ± 2.5 mmHg, *p* = 0.36). There was a significant difference in lying position (17.3 ± 2.3 mmHg versus 18.4 ± 2.5 mmHg, *p* < 0.01) and lying position with raised legs (19.3 ± 2.8 mmHg versus 18.1 ± 2.2 mmHg, *p* < 0.01). Figure 3 is a percentagewise presentation of the IOP deviation distribution.

Measuring the intergroup difference between the three measurement groups (iCare supine, iCare lying with raised legs and GAT sitting) revealed no difference between the two iCare measured groups and statistically significant difference between the GAT and the supine iCare (*p* = 0.002) and the lying with raised legs iCare (*p* < 0.001) measurements. The median value for each of the significant differences was 1 mmHg.

The Fig. 3 describes the distribution of the difference in IOP values between the prototype and the reference instrument. The graph presents the percentage of difference for each value between 0 and 6 mmHg. According to the presented results 80% of measurements were in the range of 0 + / − 1 mmHg and 90% in the range of 0 + / − 3 mmHg.

## Discussion

When comparing the results of measurements taken in the present study in all studied body positions no significant difference was observed between the results of the prototype and the reference instrument. The difference between the IOP readings in each of the three body positions was clinically insignificant.

Comparing the results by the reference instrument revealed that while measurements conducted by GAT were not significantly different from those conducted by the prototype those conducted by iCare demonstrated statistically (but not clinically) significant difference.

As a rule, the gold standard for IOP value was GAT. Icare was used in order to enable measurement in lying position. We chose lying position in order to widen the range of measurements. No difference was found between the GAT and the prototype. The average difference in the measurements by the prototype and the iCare was around 1 mmHg. Although this difference reached statistical significance it is insignificant clinically and in fact is in the range of acceptable measuring error range. The fact that the median value of the inter groups difference between the subgroups (iCare supine. iCare raised legs and GAT sitting) was 1 mmHg furtherly supports its clinical insignificance.

The good agreement of the prototype with the GAT is both logical and encouraging. Logical because the calibration was based on GAT. As the iCare’s reading are statistically different from those of the GAT and the prototype fully agrees with the GAT it is expected that the prototype will differ from the iCare. Encouraging because the GAT is the gold standard and full agreement with it is by far the most meaningful comparative result of the present study.

In summary, no difference between the prototype measurement and the GAT’s measurement was found. The statistically significant difference between the prototype and the iCare measurements is clinically insignificant. The results of the present report support the proposed prototype as possible reliable mean for non–contact and self-performed IOP measurement. Due to the limited number of participants and the fact that no high IOP measures were included in the present study further large-scale studies are needed to support the conclusions of the present study and establish the role of the proposed prototype as a useful tool in the management of glaucoma patients.

## Supplementary Information

Below is the link to the electronic supplementary material.

Supplementary file1 (TIFF 1362 KB)
