# Supplementary figures and images for: Accuracy of a novel method for IOP measurement without applying pressure on the cornea
Source: Int Ophthalmol. 2024 Jul 1;44(1):300. doi: 10.1007/s10792-024-03213-7 (PMC11217116; doi:10.1007/s10792-024-03213-7)

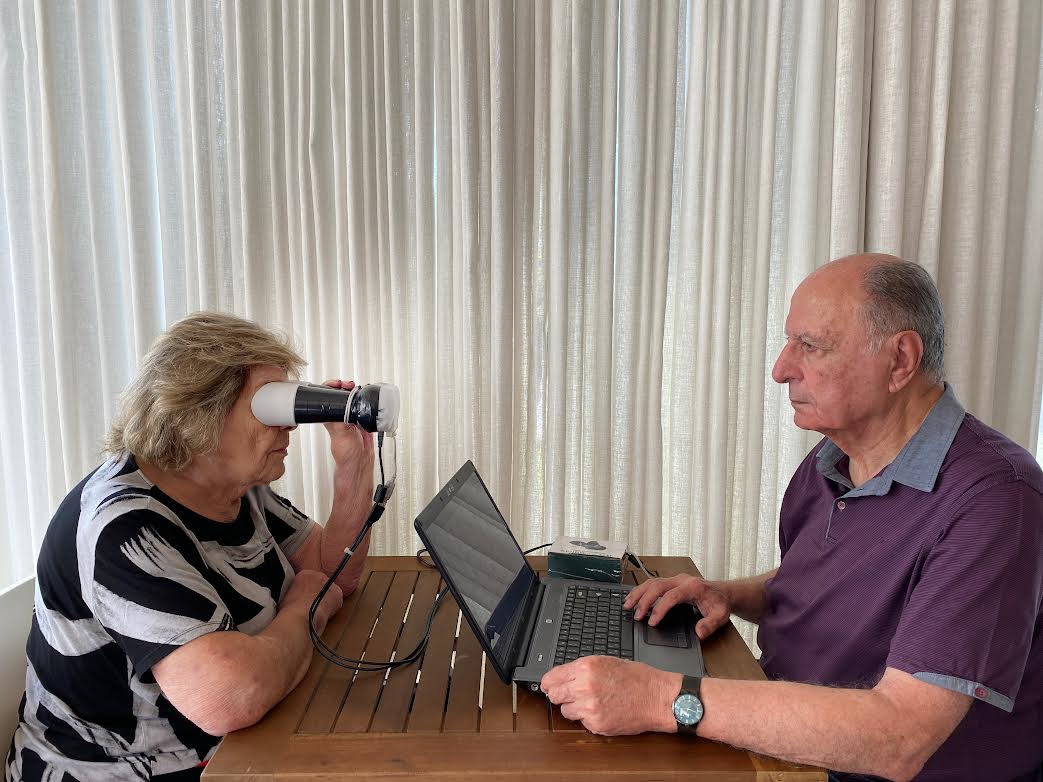

Supplement: Supplementary file 1 — Supplementary file1 (TIFF 1362 KB) [file 10792_2024_3213_MOESM1_ESM.tiff]
